# The Human *TET2* Gene Contains Three Distinct Promoter Regions With Differing Tissue and Developmental Specificities

**DOI:** 10.3389/fcell.2019.00099

**Published:** 2019-06-07

**Authors:** Hong Lou, Hongchuan Li, Kevin J. Ho, Luke L. Cai, Andy S. Huang, Tyler R. Shank, Michael R. Verneris, Michael L. Nickerson, Michael Dean, Stephen K. Anderson

**Affiliations:** ^1^Laboratory of Translational Genomics, Frederick National Laboratory for Cancer Research, Gaithersburg, MD, United States; ^2^Basic Science Program, Frederick National Laboratory for Cancer Research, Frederick, MD, United States; ^3^Cancer and Inflammation Program, Center for Cancer Research, National Cancer Institute, Frederick, MD, United States; ^4^Department of Pediatrics, Center for Cancer and Blood Disorders, University of Colorado Denver, Denver, CO, United States; ^5^Laboratory of Translational Genomics, Division of Cancer Epidemiology and Genetics, National Cancer Institute, Gaithersburg, MD, United States

**Keywords:** human, TET2, alternative promoters, demethylation, differentiation, mRNA isoforms

## Abstract

Tet methylcytosine dioxygenase 2 (*TET2*) is a tumor suppressor gene that is inactivated in a wide range of hematological cancers. TET2 enzymatic activity converts 5-methylcytosine (5-mC) into 5-hydroxymethylcytosine (5-hmC), an essential step in DNA demethylation. Human TET2 is highly expressed in pluripotent cells and down-regulated in differentiated cells: however, transcriptional regulation of the human *TET2* gene has not been investigated in detail. Here we define three promoters within a 2.5 kb region located ∼ 87 kb upstream of the first *TET2* coding exon. The three promoters, designated as Pro1, Pro2, and Pro3, generate three alternative first exons, and their presence in *TET2* mRNAs varies with cell type and developmental stage. In general, all three *TET2* transcripts are more highly expressed in human tissues rich in hematopoietic stem cells, such as spleen and bone marrow, compared to other tissues, such as brain and kidney. Transcripts from Pro2 are expressed by a broad range of tissues and at a significantly higher level than Pro1 or Pro3 transcripts. Pro3 transcripts were highly expressed by embryoid bodies generated from the H9 ES cell line, and the major Pro3 transcript is an alternatively spliced mRNA isoform that produces a truncated TET2 protein lacking the catalytic domain. Our study demonstrates distinct tissue-specific mechanisms of *TET2* transcriptional regulation during early pluripotent states and in differentiated cell types.

## Introduction

DNA methylation plays a critical role in regulating gene expression during development. The discovery of the ten-eleven translocation (*TET*) gene family has significantly impacted the field of epigenetics (Ooi and Bestor, 2008; Tahiliani et al., 2009; Ito et al., 2010, 2011; He et al., 2011; Koh et al., 2011). The TET enzymes, including TET1, TET2, and TET3, are methylcytosine dioxygenases that convert 5-methylcytosine (5-mC) to 5 hydroxymethylcytosine (5-hmC), 5-formylcytosine (5-fC), and 5-carboxycytosine (5-caC), leading to excision repair and replacement by a unmethylated cytosine, resulting in demethylation and gene activation. DNA demethylation can also occur as a result of replication-mediated dilution, since oxidized 5-mC is not recognized by the DNMT1 maintenance methylase (Valinluck and Sowers, 2007; Hashimoto et al., 2012). The TET2 protein plays an important role in the epigenetic regulation of gene expression during embryogenesis (Dawlaty et al., 2013), differentiation of hematopoietic cells (Ko et al., 2011), cancer development (Ito et al., 2010; Ko et al., 2010; Zhang et al., 2010; Meisel et al., 2018), and it is involved in somatic cell reprogramming (Doege et al., 2012; Costa et al., 2013). A recent study demonstrated that disruption of *TET2* promotes the immunotherapeutic efficacy of chimeric antigen receptor T cells (Fraietta et al., 2018). In the adult, TET2 shows a broader tissue expression pattern compared to TET1 (Lorsbach et al., 2003). TET1 and TET3 contain an N-terminal CXXC domain that binds to CpG islands, whereas this domain is not present in the *TET2* gene due to a chromosomal inversion that has resulted in the formation of a distinct gene, *CXXC4* that contains the CXXC domain (Ko et al., 2013; Liu et al., 2013; Jin et al., 2016). The CXXC4 (IDAX) protein binds to CpG islands and recruits TET2, resulting in the latter’s degradation. A similar mechanism was demonstrated for the TET3 CXXC domain, indicating a negative regulatory function for these CXXC domains. Interestingly, TET1 and TET3 isoforms lacking the CXXC domain also exist (Melamed et al., 2018). Despite numerous studies on TET family members via loss- and gain-of-function approaches, the transcriptional mechanisms underlying tissue-specific expression of the human gene have not been fully explored (Ito et al., 2010; Wu et al., 2011; Pan et al., 2017; Yang et al., 2018). The study of transcriptional regulation is fundamental to our understanding of how gene expression and phenotype are controlled during development. The expression of a given gene can be exquisitely complex, due to the presence of multiple transcription start sites that drive the expression of alternative mRNA isoforms (Djebali et al., 2012).

We define three distinct promoter elements associated with three separate clusters of transcription start sites (TSS) within a ∼2.5 kb region of the human *TET2* gene, and demonstrate cell-specific promoter activity through reporter assays in cell lines. Each promoter exhibits distinct characteristics, and the level of transcription from each promoter varies among pluripotent and differentiated cell types. The major transcript produced by the central Pro2 promoter is broadly expressed and is present at a significantly higher level than other transcripts in normal human tissues. In contrast, Pro1 transcripts are most abundant in spleen, and Pro3 transcripts are enriched in tissues that possess a greater proportion of progenitor cells. These findings may lead to new insights into our understanding of the pathogenesis of tumors and facilitate the development of novel approaches to the prevention or diagnosis of cancer.

## Materials and Methods

### Computational Analysis of the *TET2* Gene

The full sequence surrounding the *TET2* gene was obtained through the Ensembl database and the UCSC genome browser for human, mouse and rat. Identification and sequence analysis of evolutionarily conserved regions (ECRs) of *TET2* were performed with the ECR Browser, and the publicly available web-based tool mVISTA (Frazer et al., 2004) using the MLAGAN algorithm. A search for potential transcription factor binding sites in the upstream regulatory region of a particular *TET2* gene was performed online using the MatInspector program (Genomatix, Germany).

### Cell Culture and Tissues Samples

Cancer cell lines were obtained from the American Type Culture Collection (Manassas, VA, United States) and maintained according to the supplier’s instructions. The human H9 ES cell line was grown on irradiated CF-1 mouse embryo fibroblasts. Embryoid Bodies (EB) were generated by plating 3000 H9 cells/well in a 96-well plate in BPEL media supplemented with recombinant human Bone Morphogenic Protein 4 (20 ng/uL), recombinant human Vascular Endothelial Growth Factor (20 ng/uL), and recombinant human Stem Cell Factor (40 ng/ul) for 14 days.

A panel of 20 normal human tissues was purchased from OriGene Technologies (Rockville, MD, United States). Human prostate tissue samples were obtained from OriGene, John Hopkins University (Baltimore, MD, United States), Ambion (Foster City, CA, United States), and Clontech Laboratories (Mountain View, CA, United States).

### RNA Isolation, cDNA Synthesis, and Real-Time Quantitative RT-PCR

Total RNA extracted from NCI 60 cancer cell lines was received from the Developmental Therapeutics Program (DTP), Information Technology Branch, NCI. RNA was further purified and cDNA synthesis was performed. Total RNA of cells was extracted and purified as described (Lou et al., 2009). cDNA was synthesized using the Transcriptor First Strand cDNA Synthesis Kit (Roche) with oligonucleotide (dT)_18_ primer according to the manufacturer’s instructions. TaqMan^®^ Gene Expression Assay primer and probe (FAM-labeled) sets (Applied Biosystems, Foster City, CA, United States) for *TET2* (Hs00969056_m1) were used to quantify *TET2* mRNA and the results were normalized to the *HPRT1* (Hs02800695_m1) housekeeping gene by the delta CT method. Relative mRNA expression levels of the target genes were calculated with a human universal control (HUC from Clontech) as 100%.

### Generation of Luciferase Reporter Plasmids

A series of truncated *TET2* promoter constructs, including 5 deletions from the 5′ side and 3 deletions on the 3′ side, were created by PCR using the primers shown in Table 1. PCR products were cloned into the TOPO-TA vector. Inserts were excised with SacI and XhoI and cloned into the pGL3 vector (Promega, Madison, WI, United States) to generate constructs in the forward orientation. All subclones were verified by sequencing. Sequence analysis was performed with the Molecular Evolutionary Genetics Analysis (MEGA) software version 7.

**Table 1:** Gene-specific primers used for the TET2 gene.

Primer	Orientation	Sequence (5′- > 3′)	Position*	Length (bp)
**PGL3 Constructs**				
TET2-Prol-For	Forward	CACTCTTCAAAGAGGAAATATTCC	-633	684
TET2-Prol-Rev	Reverse	TGGGAAATTGACCGAGTTC	+51	
TET2-Pro2-For	Forward	CCCAGGTCTCCACTGCGC	+614	411
TET2-Pro2-Rev	Reverse	CTTGCTTCCTTCTGCTTCTC	+1,024	
TET2-Pro3-For	Forward	CTCCCTTTCTTCTCCCCTTC	+1,136	479
TET2-Pro3-Rev	Reverse	TCACAAACCCAGACCCAGACC	+1,614	
**TET2-Pro RT-PCR**				
TET2-Prol-RT-For	Forward	AGATAGAGACGCGGGCCTCTGAGG	+189	
TET2-Pro2-RT-For	Forward	GCAAGGCTGAGGGACGAGAACGAG	+1,082	
TET2-Pro3-RT-For	Forward	CAGCCTGGGGAATGTATGTAAGAG	+1,837	
TET2-Pro-RT-Rev6	Reverse	CTCITCACTGCTGCITCTGCGAACC	TET2 Exon 6	
TET2-Pro-RT-Rev3	Reverse	GGGAGGTGATGGTATCAGGAATGG	TET2 Exon 3	


*Relative to the TET-1a transcription start site.

### Cell Transfection and Luciferase Assays

The breast cancer cell line MCF7, cervical cancer cell line HeLa, prostate cancer cell line PC3, trophoblast cell line JAR, human embryonic kidney 293T cell line, and pluripotent human embryonal carcinoma cell line Ntera-2 were used for the analysis of promoter constructs. The cells were plated at 1 × 10^5^ cells per well in a 24-well plate the day before transfection and incubated overnight at 37°C in 5% CO2. For each well, 5 μL of HilyMax transfection reagent (Dojindo, Rockville, MD, United States) was diluted in 30 μL of growth medium without serum containing 1 μg of the specific reporter construct with 5 ng of Renilla luciferase pRL-SV40 control DNA and incubated at room temperature for 5 min. The DNA transfection mixture was then added to each well and incubated at room temperature for 20 min. Luciferase activity was assayed at 48 h using the Dual-Luciferase Reporter Assay System (Promega) according to the manufacturer’s instructions. Measurements of the firefly luciferase activities of the TET2 promoter constructs were normalized relative to the activity of Renilla luciferase produced by the pRLSV40 control vector. Each construct was tested in triplicate in at least three independent experiments.

### Western Blot Analysis

Whole cell protein lysates were prepared from the cell lines by using RIPA lysis buffer containing complete Protease Inhibitor Cocktail (Santa Cruz, CA, United States) according to the manufacturer’s protocols. Seventy five micrograms of whole cell protein were separated in a 3–8% NuPAGE Tris-Acetate gel, transferred to PVDF membrane (Thermo Fisher Scientific, Carlsbad, CA, United States), and then immunoblotted with the primary antibodies in 0.05% Tween 20-Tris-buffered saline (TBST) containing 5% skim milk at 4°C with shaking overnight. The primary antibodies used were: a mouse monoclonal anti-TET2a that recognizes the amino terminus of TET2, (C15200179, Diagenode) at a dilution 1:1,000 and a polyclonal rabbit-anti-TET2b (R1086, Abiocode) that recognizes the C-terminus of the short isoform of TET2, at 1:800. The β-actin, used as an internal control, was detected by rabbit-anti -β-actin polyclonal antibody (Cell Signaling Technology, MA, United States). HPR-conjugated anti-rabbit and mouseIgG (1:8,000, Cell Signaling Technology) and anti-rabbit IgG (1:3000, Cell Signaling Technology, MA, United States) were used as secondary antibodies. The membranes were incubated with SuperSignal West Pico peroxide and luminal enhancer solutions (Pierce, IL, United States) for 5 min, exposed to the film and developed.

### Statistical Analysis

Mann-Whitney-U and two-tailed *t*-test were performed using GraphPad Prism version 7 for Mac OS; *p* < 0.05 was regarded to be statistically significant.

## Results

### Identification of Three Distinct Human *TET2* Promoters

**FIGURE 1:**
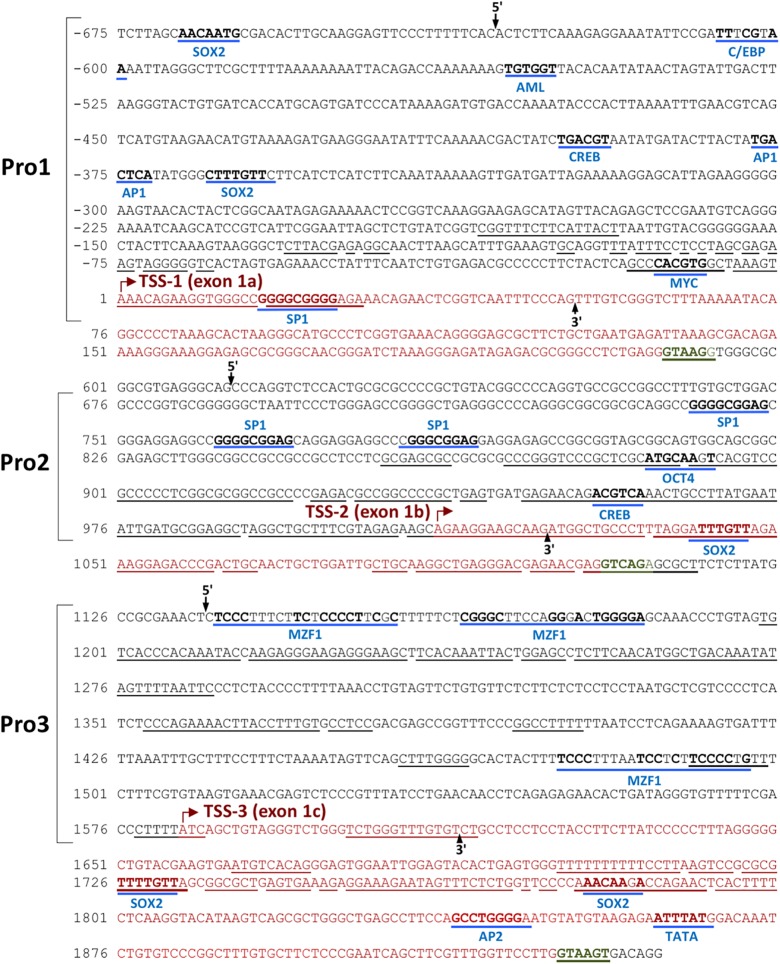
Identification of three *TET2* promoter elements. The sequence of the 2.5 kb region containing the three *TET2* promoters is shown. The sequence is numbered relative to the Pro1 transcript start site. Predicted transcription factor binding sites are underlined in blue, with consensus nucleotides denoted in bold. The transcription starting sites (TSSs) are marked with a red arrow, and the corresponding exon sequence is shown in red type, ending at a consensus splice donor sequence in green type. Sequence conserved in the mouse *TET2* gene is underlined. The proposed promoter regions are indicated by the brackets, and the 5′ and 3′ ends of each promoter construct tested in luciferase reporter assays are indicated by labeled arrows.

**FIGURE 2:**
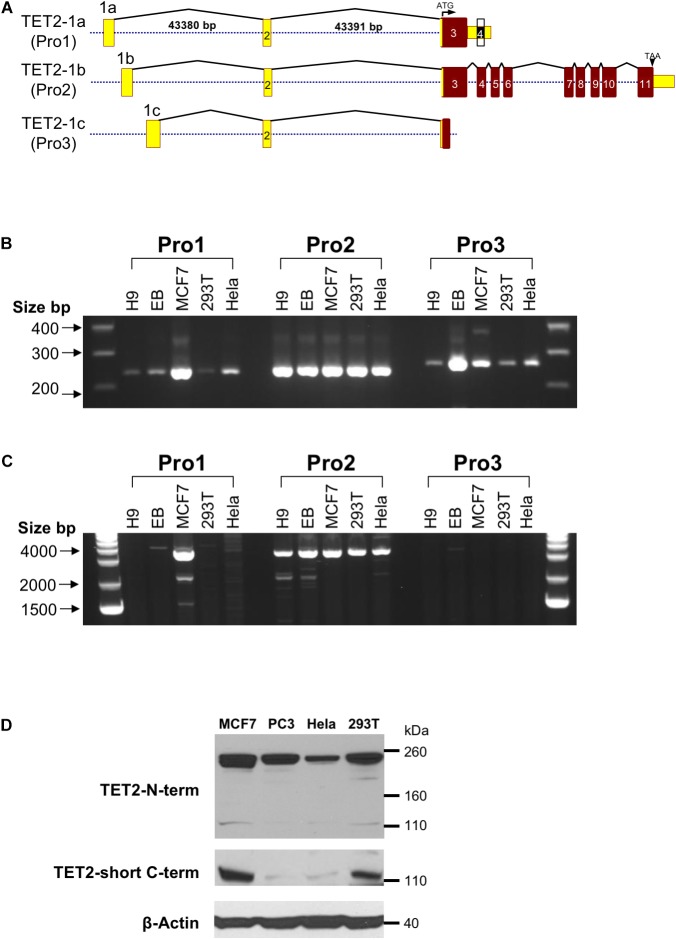
Observed *TET2* transcripts and proteins **(A)** Schematic representation of the exon-intron organization of the three distinct transcripts of the human *TET2* gene observed in GenBank. Exons are indicated by numbered rectangles, and the size of the first and second introns is shown for the TET-1a transcript. The translated region and untranslated region (UTR) are indicated in red and yellow boxes, respectively. The structure of the largest cDNA clone observed for each transcript is shown. **(B)** RT-PCR with Pro1, Pro2, or Pro3-specific forward primers and an exon 3 reverse primer. **(C)** RT-PCR with Pro1, Pro2, or Pro3-specific forward primers and an exon 6 reverse primer. **(D)** Western blot of TET2 proteins with antisera raised against either the amino terminal region of TET2 (TET2 N-term) or the unique C-terminus of the truncated TET2 protein (TET2-short C-term) were used to probe lysates from the MCF7, PC3, HeLa, and 293T cell lines. Anti-β-actin was used as a loading control.

*In silico* promoter region prediction and gene analysis were performed using the Genomatix ElDorado and Gene2Promoter online programs (Genomatix), together with a visual inspection of the 5′ regulatory region and reported transcripts using the UCSC Genome Browser^1^. Multiple transcription start sites associated with three alternative first exons were identified within a 1,891 bp region of *TET2*, and the nucleotide sequence has been numbered relative to the first nucleotide of the 5′-most TSS in Figure 1. Three predicted promoter regions (Pro1, Pro2, and Pro3) that produce three alternative first exons (1a, 1b, and 1c) are present within the 2,612 bp region shown, and likely play a role in the regulation of *TET2* expression in various cell types (Figure 1). Only a single 10 kb full-length cDNA originating from the Pro2 promoter region (GenBank# FM992369) that contains the complete 2002 amino acid (aa) TET2-coding region has been reported (TET2-1b; Figure 2A). A single 9 kb Pro1 transcript isolated from human fetal kidney has been reported (GenBank# BX640738) that terminates at a polyA site in the fourth intron, resulting in a truncated 1165 aa open reading frame that lacks the C-terminal TET2 catalytic domain (TET2-1a). Only partial Pro3 cDNAs terminating in exon 3 have been identified (GenBank#s DA495712 and DA441067). In order to determine if Pro1 and Pro3 transcripts extend beyond the polyA site in the fourth intron, RT-PCR was perfomed with exon 1a, 1b, and 1c forward primers together with reverse primers in either exon 3 or exon 6. All three exon 1 forward primers successfully amplified products with an exon 3 reverse primer (Figure 2B). However, transcripts extending to exon 6 were only detected with Pro1 (exon 1a) and Pro2 (exon 1b) forward primers, suggesting that Pro3 transcripts do not produce any catalytically active TET2 protein (Figure 2C). In order to determine if the predicted short TET2 isoform lacking the catalytic domain is expressed, a Western blot analysis was performed using antibodies raised against either the amino-terminus present in both isoforms, or the carboxy-terminus specific to the predicted short form (TET2-1a; Figure 2A). The short isoform was found to be expressed in all of the cell lines, with the highest expression in MCF7 and 293T cells (Figure 2D), consistent with the detection of Pro1 or Pro3 transcripts in these cell lines (Figure 2B). The amino-terminal TET2 antibody also detected a faint lower molecular weight band corresponding to the size of the short form (upper panel, Figure 2D). The weak expression of the truncated TET2 protein relative to the full-length protein suggests that the majority of *TET2* transcripts in these cell lines contain the full coding sequence.

### Functional Analysis of the Human *TET2* Promoters

**FIGURE 3:**
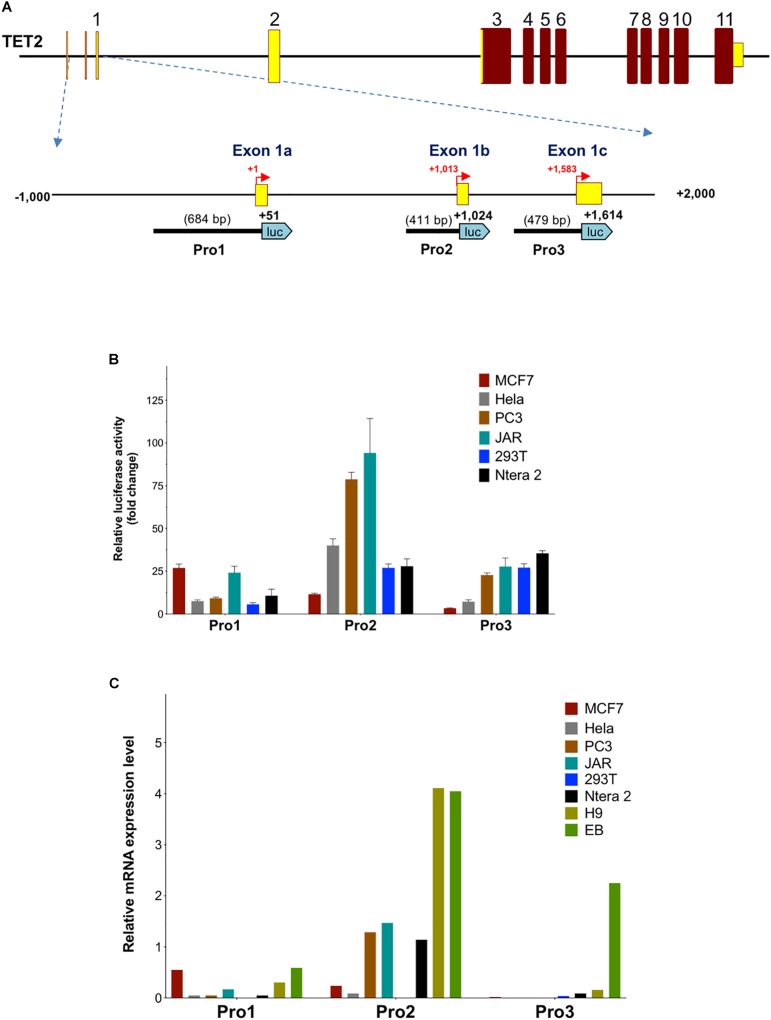
Activity of *TET2* promoters in cell lines. **(A)** A schematic of the *TET2* gene structure is shown with an expanded view of the 3 kb regulatory region. The relative sizes and positions of fragments cloned into the pGL3 vector are indicated by the lines below the schematic. The nucleotide position of the 3′ end is indicated in bold, and the insert size is shown in parentheses. **(B)** The promoter activities measured after transfection into MCF7, HeLa, PC3, JAR, 293T, and Ntera-2 cells are shown. The luciferase activity of each pGL3 construct is shown as fold-increase of corrected light units relative to an empty pGL3 vector control. Values represent the mean and error bars indicate the SEM of at least three independent experiments. **(C)** Relative Pro1, Pro2, and Pro 3 mRNA levels in cell lines. Total RNA from the cell lines used in **(B)** together with H9 ES cells and embryoid bodies (EB) was reverse-transcribed, and transcript levels were investigated by qPCR relative quantification analysis with Pro1, Pro2, and Pro3-specific primers. The cDNA levels of *TET2* Pro1, Pro2, and Pro3 were normalized to β-actin.

In order to investigate the properties of the multiple promoter elements in the 5′-flanking region of *TET2*, three DNA fragments containing the Pro1 promoter region (-633 to +51 bp), Pro2 promoter region (+614 to +1,024 bp), and Pro3 promoter region (+1,136 to +1,634 bp) were cloned into the pGL3 vector (Figure 3A), and the promoter activities were determined in MCF7, HeLa, PC3, JAR, 293T, and Ntera-2 cells. As shown in Figure 3B, the highest promoter activity was observed with Pro2 constructs in PC3 and JAR cells: 75-fold and 90-fold relative to empty pGL3 control vector, respectively. The transcriptional activity of the 410 bp *TET2* Pro2 core promoter construct was significantly higher than that of the Pro1 and Pro3 fragments in most of the cell lines tested (Figure 3B). These results are consistent with the observation that the majority of *TET2* transcripts observed originate from the Pro2 region (see text footnote 1). Interestingly, the Pro1 and Pro3 elements displayed distinct cell type specificities: Pro1 activity was significantly higher in MCF7 and JAR cells than any of the other lines tested, whereas Pro3 activity was lowest in MCF7 cells and higher in PC3, JAR, 293T, and Ntera2 cells. Q-PCR with primers specific for the Pro1, Pro2, or Pro3 transcripts was performed to determine if transcript levels correlated with promoter activity in these lines. Pro1 transcript levels were highest in MCF7 and JAR cells, consistent with the luciferase results (Figure 3C). Pro2 transcript levels were high in PC3 and JAR cells, in agreement with their Pro2 promoter activity: however, Hela and 293T did not have transcript levels that matched their *in vitro* promoter activity. Pro3 transcripts were low to undetectable in the cell lines studied, therefore embryoid bodies (EB) were generated from the human H9 ES cell line to generate a population enriched for progenitor cells. Remarkably, Pro3 transcripts were much higher in EB than any of the cell lines tested, including the H9 ES cell line from which they were generated, suggesting that Pro3 represents a progenitor cell-specific promoter. Taken together, these results are consistent with Pro2 functioning as a strong, ubiquitous promoter, and Pro1/Pro3 functioning in differing subsets of progenitor cells and cancer cell lines.

### *TET2* mRNA Expression Levels in Cancer Cell Lines

**FIGURE 4:**
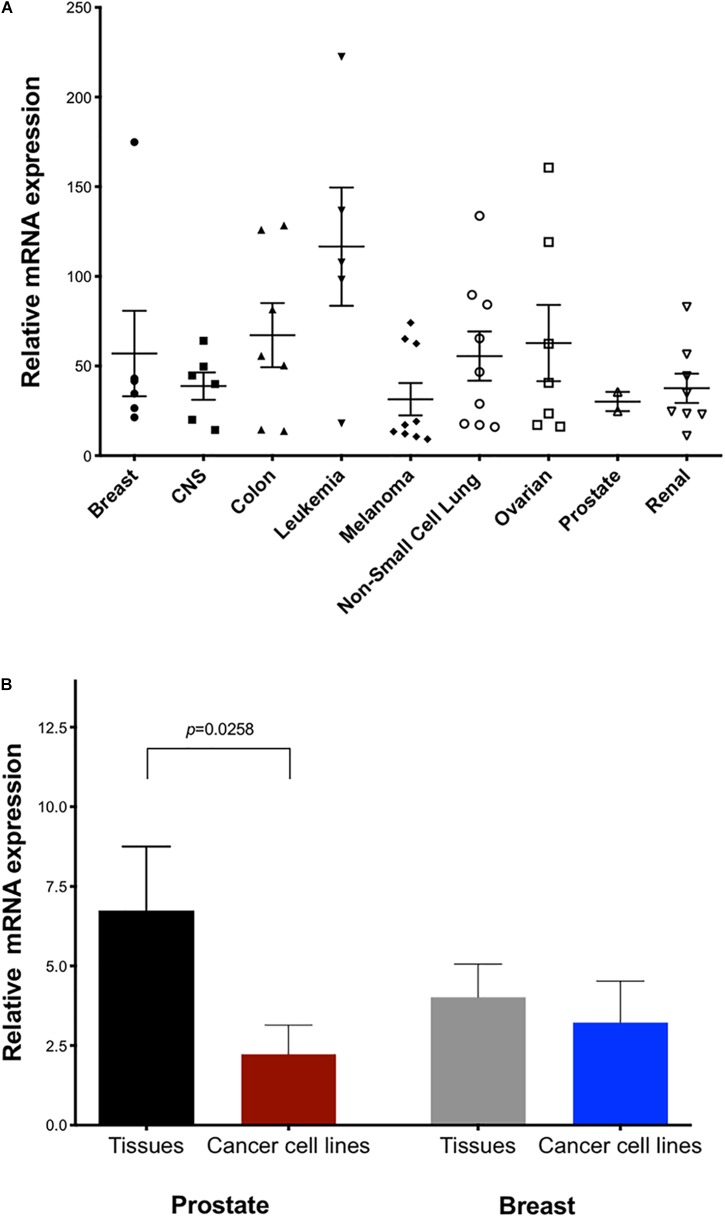
Expression of TET2 in cancer cell lines and tumor tissue. **(A)** Expression of human *TET2* in the NCI-60 cancer cell line panel was measured by qPCR. The expression level was determined relative to a human universal control (HUC from Clontech) as 100%. HPRT1 was used as the housekeeping gene **(B)** Expression levels of TET2 in human tissue RNA panels and cancer cells from prostate and breast are shown.

The NCI-60 human cancer cell line panel represents a wide array of solid tumors and leukemias. We measured *TET2* mRNA levels in the panel using the TaqMan real-time PCR method with primers targeting exons 6 and 7 (Figure 4A). Cell lines expressing high levels of *TET2* mRNA were observed for most tissues, perhaps reflecting involvement in conserved biological or cancer-relevant processes. Notably, there are cell lines with either high or low *TET2* expression in the cancer cell lines from most tissues, with the exception of the prostate lines. These high and low classes of *TET2* expression may reflect the progenitor/differentiation potential of the individual lines. A comparison of *TET2* transcripts between normal and malignant prostate and breast tissues revealed significantly lower levels of *TET2* mRNA in prostate cancer cell lines as compared to normal tissue, but not in breast cancer cell lines, suggesting that downregulation of TET2 may be a common event in prostate cancer (Figure 4B).

### Quantitation of *TET2* mRNA Isoform Expression Levels in Human Tissue

**FIGURE 5:**
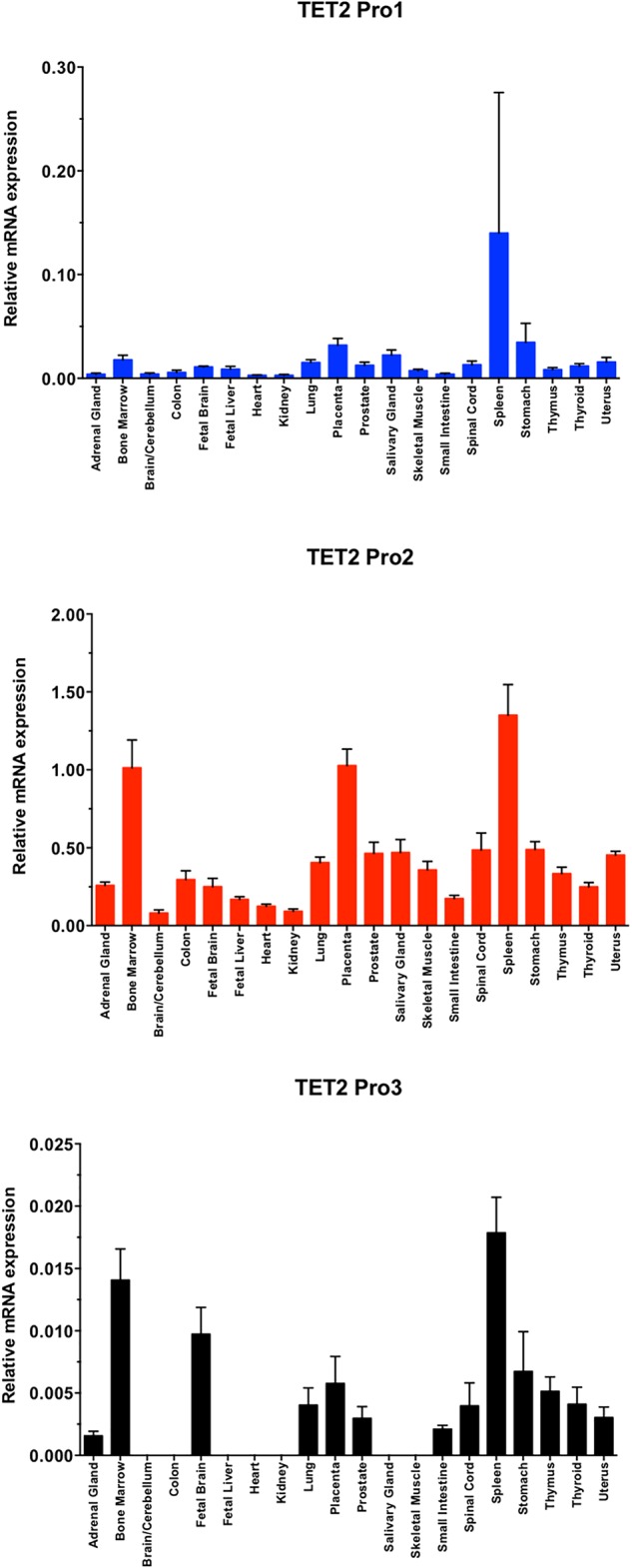
TET2 transcript levels in normal tissues. A total RNA from a human tissue RNA panel was reverse-transcribed, and cDNAs were investigated by qPCR relative quantification analysis with Pro1, Pro2, and Pro3-specific primers. Tissues of origin are listed for each lane. The cDNA levels of TET2 Pro1, Pro2, and Pro3 were normalized to β-actin.

To further investigate the tissue specificity of the three *TET2* promoters, the major *TET2* Pro2, less abundant Pro1, and rare Pro3 transcript levels were analyzed in 20 human normal tissues by RT-qPCR (Figure 5). The major Pro2 transcripts showed broader tissue expression and significantly higher expression levels than Pro1 and Pro3 in all 20 normal human tissues, whereas the Pro1 and Pro3 showed more restricted tissue-specific expression.

## Discussion

In the present study, we define three promoters contained within a 1583 bp region of the *TET2* gene with distinct tissue- and developmental stage-dependent transcriptional activities. The Pro2 promoter had the strongest activity in all cell lines tested, with the exception of MCF7, in which Pro1 showed the greatest activity (Figure 3B). The dominant promoter activity of Pro2 is supported by a survey of reported *TET2* mRNAs performed using the UCSC Genome Browser that reveals the majority of *TET2* transcripts originate from the Pro2 region. Measurement of *TET2* Pro1, Pro2, and Pro3 transcript levels in a panel of 20 normal human tissues confirmed that Pro2 transcripts were the most abundent in normal human tissues. A direct comparison of transcript levels in total spleen RNA revealed that Pro2-derived transcripts are 10-fold higher than Pro1 transcripts, and 100-fold higher than Pro3 transcripts (Figure 5). The broader tissue expression and significantly higher level of Pro2 transcripts than Pro1 and Pro3, suggests that this promoter is less dependent on pluripotency.

In contrast, Pro1 is active primarily in human spleen, whereas the rare Pro3 transcript was weakly expressed in most of the tissues tested, with the highest levels observed in human spleen, bone marrow, and fetal brain. The more restricted expression of Pro1 and Pro3 transcripts relative to Pro2 transcripts (Figure 3C, 5), suggests that the TET2 isoforms produced from these transcripts may play an important role in hematopoiesis and stem cell differentiation. It is of particular interest that ES cells differentiated into embryoid bodies show increased transcription from Pro3 (Figure 3C), and that Pro3 transcripts do not contain the full coding region (Figure 2C). Perhaps the truncated TET2 protein lacking the catalytic domain acts as a dominant negative protein that inhibits TET2-mediated demethylation in progenitor cells. Our study provides the groundwork for further investigation of the mechanisms controlling the expression of human *TET2* isoforms.

Recently, Sohni et al. (2015) defined promoter, enhancer, and super-enhancer regions in the murine *TET2* gene and investigated their activity in differentiating embryonic stem (ES) cells. A single *TET2* promoter was identified that corresponds to the human Pro2 element, and ES cell transcription was associated with a conserved element in the downstream super-enhancer region. However, an upstream transcript originating in the Pro1-homologous region of the murine *TET2* gene has been observed in a spleen EST clone (GenBank#BY223169), and the promoter activity of this region was not investigated by Sohni et al. (2015). Interestingly, three distinct promoters and alternative first exons were identified in the murine *TET1* gene (Sohni et al., 2015). The three *TET1* promoters bear no homology to the three *TET2* promoters identified in the current study, since the 5′ end of the *TET2* gene lacks the CXXC domain containing exons found in *TET1*, and therefore is in a distinct genomic region. Alignment of the human and mouse *TET2* genes reveals conservation of the regions within the Pro1, Pro2, and Pro3 promoter regions (Figure 1). The greatest blocks of homology are within the Pro2 region. The broad tissue specificity and dominant promoter activity of Pro2 is conserved between human and mouse. The more limited homology of the Pro1 and Pro3 regions suggests that the function of these elements may not be conserved between species. The *TET2* Pro3 element may represent a stem cell-specific promoter that supports high levels of TET2 expression in progenitor cells. It contains multiple predicted MZF1-binding sites that have been associated with stem cell promoters, such as the CD34 and KIR-antisense promoters (Morris et al., 1995; Wright et al., 2013). MZF1 was found to be expressed in the 293T cell line (Wright et al., 2013), and Pro3 promoter activity was highest in 293T cells (Figure 3B). Pro3 transcripts were highest in bone marrow, fetal brain, spleen, and embryoid bodies (Figure 3, 5), consistent with progenitor-specific activity. The study of *TET2* transcripts in murine pluripotent cells did not reveal any Pro3-like element (Sohni et al., 2015), and the predicted MZF1-binding elements are not conserved in the murine gene (Figure 1), suggesting that the human and mouse genes have distinct mechanisms to drive TET2 expression in progenitor cells.

Since the TET2 CXXC regulatory domain is a separately transcribed gene, *CXXC4*, it is of interest to consider if there are particular tissues or developmental stages where either TET2 or CXXC4 (IDAX) expression dominates, changing the rate at which TET2-mediated demethylation occurs. *CXXC4* mRNA levels in murine ES cells increase when they are induced to differentiate, leading to a dramatic loss of TET2 protein without significantly affecting *TET2* mRNA levels (Ko et al., 2013). Conversely, loss of CXXC4 expression has been observed in aggressive renal cell carcinomas, and is associated with reduced survival, which may be due to an increase in cancer stem cells (Kojima et al., 2008).

**FIGURE 6:**
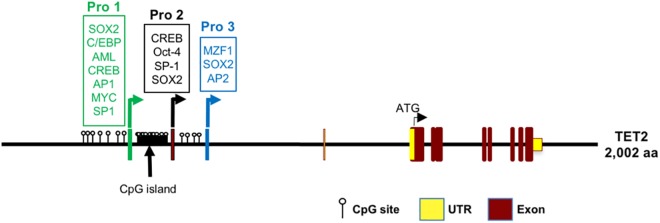
Model of transcription regulation of the *TET2* gene based on this study. Pro1 (exon -1a promoter), Pro2 (exon -1b promoter), and Pro3 (exon -1c promoter) with putative transcription factor binding sites are shown. The position of CpG dinucleotides in the TET2 regulatory region are indicated.

Considered together, the results suggest that multiple promoter elements have evolved in the human *TET2* gene to allow *TET2* gene transcriptional regulation to be controlled differently in pluripotent cells, stem cells, and differentiated tissues (Figure 6). The precise physiological relevance of human TET2 expression in different cell states will require further exploration. The results provided in this study are applicable to studies of *TET2* in human cancers and may explain some of the previous SNP associations, such as rs7679673 of *TET2* correlating with a family history of prostate cancer (Eeles et al., 2009; Haiman et al., 2011; Nickerson et al., 2013; Al Olama et al., 2014). The distinct properties of the three *TET2* promoter elements may play an important role in human cancer development and differentiation and should provide new insights into understanding pathogenesis and development of new therapeutic approaches.

## Data Availability

All datasets generated for this study are included in the manuscript and/or the supplementary files.

## Author Contributions

HLo, HLi, MD, and SA contributed to the conception of the study. HLo and HLi contributed to the design of the study. HLo, HLi, KH, AH, TS, and LC performed the experiments. HLo, HLi, and SA analyzed the data. HLo, HLi, LC, MV, MD, and SA wrote and edited the manuscript. All the authors contributed to manuscript editing, and approved the submitted version.

## Conflict of Interest Statement

The authors declare that the research was conducted in the absence of any commercial or financial relationships that could be construed as a potential conflict of interest.

## Footnotes

1 http://genome.ucsc.edu
